# Revisiting Handwriting Fundamentals Through an Interdisciplinary Framework

**DOI:** 10.21315/mjms2022.29.1.3

**Published:** 2022-02-23

**Authors:** Ann Sien Sut Lee, Lay Wah Lee, Hui Min Low, Siew Chen Ooi

**Affiliations:** 1 School of Educational Studies, Universiti Sains Malaysia, Pulau Pinang, Malaysia; 2 Department of Occupational Therapy, Penang General Hospital, Pulau Pinang, Malaysia

**Keywords:** handwriting, interdisciplinary framework of handwriting, Malay language handwriting, handwriting fundamentals

## Abstract

Handwriting research lies mostly within discipline-specific boundaries, hindering knowledge transfer across disciplines into academic skills instruction in schools. This paper attempts to review the literature on handwriting across the occupational therapy and education disciplines to propose an interdisciplinary conceptual framework to guide research and intervention on handwriting in the Malay language. This cross-disciplinary review revealed four major factors that may influence Malay language handwriting: i) neuromotor development; ii) ergonomic; iii) orthographic and iv) cognitive factors. The sub-factors under these four major factors also are identified. Many of the neuromotor development and ergonomic factors are derived from the occupational therapy discipline, while the education discipline provides most of the information on orthographic and cognitive factors. As orthography influences handwriting, it is necessary to revisit handwriting from the perspective of languages other than English. In conclusion, an interdisciplinary framework of handwriting synthesised from this cross-disciplinary review will stimulate more coordinated and coherent research on handwriting. The Malay language serves as a future case study for research into orthographies in handwriting.

## Introduction

Alphabetic handwriting is a process of producing or transcribing letters to form words and sentences (1, 2), a process not to be confused with writing or composing. This review focusses on handwriting in the Malay language, which uses the 26 letters of the English alphabet. Handwriting is viewed as a lower mechanical level of writing, whereas the writing process itself is viewed as a higher-level process that involves cognitive comprehension (3). Handwriting research was popular during the 1980s and 1990s within numerous disciplines, such as neurology, psychology, education and linguistics, among others. The extensive studies conducted during that era resulted in a deeper understanding of handwriting, matured assessment methods, interventions and the development of handwriting models (1). However, by the end of the 20th century, with the emergence of typewriting, followed by the rapid development of digital writing technology, the need to learn handwriting was questioned (1).

In this technological era, we produce text in various ways, such as typewriting (keyboarding skills), digital writing (writing with electronic writing devices) and the use of speech-to-text software. Even so, a study by Mueller and Oppenheimer (4) revealed that taking notes in the traditional way is more beneficial than the use of digital devices. They found that taking notes by hand increases retention of factual content and conceptual understanding. Note-taking also encourages a more cognitive processes, thereby contributing to effective learning. Mangen et al. (5) found that handwriting helped with word retrieval more than typewriting on conventional and touch keyboards. Another study comparing learning through tablets with the traditional method also suggested that not employing the latter might deprive children of fine motor skills (6). Handwriting also has been found to be important for letter processing in the brain (7). In a functional magnetic resonance imaging study, James and Engelhardt (8) detected stimulation in the brain’s reading circuit among 5-year-old children while they were handwriting, an effect not found after a typing or tracing experience. These studies provide evidence of the importance of producing letters by hand, even in the technological era.

An additional research evidence also illustrates handwriting’s positive impact on performance across all academic learning types, such as reading, writing and language (1, 3, 8–10). When handwriting becomes automatic (effortless), writers can focus on composing and writing essays (1, 3). Handwriting also improves letter-recognition skills in reading and, therefore, language recognition itself (8). Handwriting difficulties also can be related in a statistically significant way to academic failure (11), considering that handwriting tasks account for 30%–60% of school activities in elementary school (12).

Handwriting is a complex task in which low- and high-level processes constantly are interacting (13, 14). ‘Low-level process’ entails execution of handwriting production, which involves neuromotor and ergonomic skills, whereas ‘high-level process’ refers to cognitive processes involved in handwriting. ‘Neuromotor skills’ refer to visual-motor integration (VMI), fine motor skills and gross motor ability, while ‘ergonomic skills’ refer to pencil-and-paper manipulation, such as gripping a pencil, positioning a pencil and paper, the consistency of pencil grip and pencil position. Cognitive processes entail working memory, long-term memory and executive attention. As these factors generally are studied independently, a better understanding of the relationships among them in the context of handwriting among struggling learners is needed to produce more efficient interventions. Handwriting skills are a prerequisite of the writing process, as poor handwriting skills directly influence this process by causing cognitive overload (15–19). Thus, proficient writing relies on well-developed handwriting skills (2, 20).

Initial searches were conducted in the EBSCOhost and Scopus databases using the keywords identified from a definition of ‘handwriting’ (‘handwriting’, ‘neuromotor’, ‘fine motor’, ‘handwriting legibility’, ‘handwriting speed’, ‘orthographic coding’ and ‘cognitive’). The abstracts from these papers were scanned, and the most relevant papers from the frequently cited education and occupational therapy disciplines were distilled. These papers were reviewed, further citations from them were identified and checked out, and the process was repeated — a systematic literature review method known as snowballing (21). The review results from both disciplines are integrated and discussed below.

## Towards an Interdisciplinary Approach to Handwriting Research

Handwriting has been studied quite substantially in various disciplines, particularly in the fields of education, neurodevelopment and occupational therapy. However, most extant studies are discipline-specific and remain within their own boundaries, resulting in a limited transfer of knowledge and skills into academic skills instruction in schools. Furthermore, few studies on handwriting combined perspectives from both the education and allied health (occupational therapy) disciplines. Therefore, this review was conducted to bridge the knowledge gap between education and occupational therapy disciplines, which are involved directly in handwriting problems among schoolchildren. Combining knowledge on handwriting from both fields would help facilitate more well-informed practical diagnosis and intervention to address children’s handwriting problems. In the occupational therapy literature, the emphasis is on acquisition and readiness of handwriting skills, whereas the influence from language characteristics on the handwriting task is almost non-existent. However, educators focus on functional writing and do not emphasise motor development in handwriting. Integration of research knowledge from these disciplines inevitably would generate a better understanding to help occupational therapists and educators address children’s handwriting difficulties.

In the education discipline, handwriting research has decreased as the focus has shifted to process writing, which de-emphasises handwriting (1, 22). According to Hayes and Berninger (23), handwriting is influenced mainly by orthography and phonology (letter shapes and sounds, respectively), whereas occupational therapists believe it is predominantly a motor-related skills issue (24).

Students are expected to master handwriting when they start school to partake in learning activities at school that largely involve fine motor skills (e.g. handwriting, cutting and drawing). Children in Malaysian national primary schools with the Malay language as their medium of instruction are expected to have general handwriting proficiency (the ability to hold and write with a pencil correctly, some mastery of alphabetic letters, an understanding of the concept of writing from left to right, etc.), including the ability to use handwriting to complete homework and exams. However, handwriting is not taught formally at national primary schools, leading to poor handwriting performance (legibility and speed), which might affect academic achievement (1). Graham (20) found that students’ handwriting legibility influences teachers’ assessment of their performance, i.e. students with poor handwriting legibility were found to score lower compared with those with legible handwriting despite the content of their written work. Slowness in handwriting also might lead to inability to complete writing tasks on time. These problems also are reflected in the Malaysian primary schools (25), thereby eliciting the authors’ interest in investigating Malay language handwriting in the present study. Furthermore, the Malay language’s unique characteristics add to this study’s value. The Malay language is one of the most highly consistent and transparent of alphabetic orthographies (26), which justifies an investigation, considering that extant research on handwriting mostly has focussed on opaque English language orthography.

In a widely adopted practice in many countries, schoolteachers identify and refer students with handwriting problems to an occupational therapist for handwriting intervention (27, 28). The occupational therapist examines the student’s handwriting ability based on knowledge in their discipline (underlying deficits such as fine motor, postural motor, sensory integration, sensorimotor, perceptual and/or behavioural elements, etc.) (29). Occupational therapists in Malaysia are trained in handwriting intervention and are particularly in demand among stakeholders of children with special needs (e.g. special-education teachers and parents). However, the lack of occupational therapists in South-Asian countries such as Malaysia (30) inevitably has resulted in a serious gap between needs and services. Therefore, a need exists to promote knowledge transfer across disciplines and make intensive clinical interventions over handwriting difficulties more available to general and special-education students in both special and inclusive classrooms (31).

This paper attempts to review relevant literature on handwriting across the occupational therapy and education disciplines to propose an interdisciplinary conceptual framework to guide future research and intervention on handwriting in the Malay language.

## The Malay Language’s Influence on Handwriting

Language plays an important role in handwriting (32). For example, each language’s grammatical rules dictate words’ letter arrangements. In addition, phoneme-grapheme correspondence and the number of syllables in a word can affect handwriting speed (33, 34). The grapheme and syllable also modulate the timing of motor production during handwriting skills acquisition. Kandel et al.’s (33) study on handwriting of two-syllable words found that the first syllable is produced grapheme-by-grapheme whereas the second syllable is produced as a whole unit and not grapheme-by-grapheme. Furthermore, knowledge of grapheme-phoneme correspondence (GPC) helps with retrieval of information stored in working memory.

Many previous handwriting studies have focussed on the English language, with some studies examining other languages, such as Chinese (35), Hebrew (36) and Urdu (37). However, handwriting research in the Malay language, the national language of Malaysia, is seriously lacking.

Although the Malay language uses the 26 letters of the English alphabet, there are differences in orthographic transparency and granularity. First, the Malay language is more transparent than the English language, in that grapheme-phoneme mappings are almost perfect. In addition, multi-letter graphemes are limited in Malay (26, 30, 38). The Malay language is also predominantly bi- and multi-syllabic (39, 40); therefore, revisiting handwriting fundamentals from the Malay language perspective is warranted, as Malay orthography’s transparency can inform handwriting issues in other orthographies with similar characteristics.

In other words, the Malay language’s unique characteristics add to this study’s value. The Malay language is one of the most highly consistent and transparent of orthographies in alphabetic languages (26), warranting investigation, considering that extant handwriting studies have focussed mostly on the English language, which is an opaque orthography.

## Handwriting Skills

Handwriting entails the formation of alphabetic letters by hand, which requires physical motor skills and alphabetic knowledge (41). Handwriting commonly is assessed based on legibility (quality) and speed (fluency) (1, 42, 43). Empirical studies indicate that these two components are not correlated (44, 45). In practice, we can see that legible handwriting can be produced either fluently or slowly; therefore, we can deduce that handwriting speed may not necessarily indicate good or poor handwriting quality. The factors that affect both aspects of handwriting are discussed in the following sections. Generally, both quality and speed improve as the student progresses to higher grades (46).

Handwriting is a process of coordinating multiple modality skills — including fine motor skills, language knowledge and academic readiness — requiring the intertwining of cognitive and motor processes that underlie the handwriting task (13, 43, 47, 48). To produce a text, various processes are initiated — including retrieval of correct letters or words from memory, arrangement of letters in the right order, conversion of phonemes to graphemes to letters, and selection and execution of corresponding motor processes (48) — depending on the handwriting mode (e.g. copying, spelling, dictating).

As the core providers of handwriting remediation and assessments, occupational therapists focus more in-depth on the lower order of writing, comprising neuromotor skills involved in handwriting (29, 43, 49, 50). According to occupational therapists, both intrinsic factors (in-hand manipulation, bilateral integration, motor planning, VMI, visual perception, kinaesthesia, sensory awareness and sustained attention) and extrinsic factors (environmental factors such as lighting, noise, distance when copying, biomechanical ergonomic factors, pencil grip, the writing instrument used, type of paper used and its placement on the desk) affect handwriting (43, 51). These intrinsic and extrinsic factors should be studied to gain a clearer insight on this subject.

Research from the education perspective provides the overall big picture of writing and views handwriting as a process that comprises only lower-level writing skills (52). Hayes and Berninger (23) proposed a cognitive framework for writing that described the comprehensive writing process from the perspective of educational psychology. According to the model (23), handwriting is a lower-level skill in the overall framework of writing, which is positioned in transcription, a subcomponent in the translation process (3). These low-level developmental skills are more important for beginning writers compared with mature ones (53). According to Berninger et al. (3), two lower-level writing processes in the transcription subcomponent (one of the subcomponents in translation; see [15] and [23]) are handwriting and spelling. However, it is worth noting that this review focusses on handwriting fundamentals, which do not include spelling, although both are viewed as lower-level writing processes.

It can be said that knowledge from both disciplines is complementary, so an interdisciplinary conceptual framework for handwriting would benefit teachers, who generally are not familiar with knowledge from the occupational therapy discipline.

## Neuromotor Developmental Factors in Handwriting

Neuromotor components related to handwriting may include fine motor skills (in-hand manipulation, bilateral integration and motor planning), VMI, visual perception, kinaesthesia and proprioception, sensory modalities and sustained attention (29, 42). Most studies on handwriting-related motor developments focus on VMI, fine motor skills and gross motor ability, which are important in the development of the ability to control a writing tool, thereby allowing for good handwriting (27, 29, 54, 55).

### Visual-Motor Integration

VMI plays a prominent role in the copying task. When a child copies a word or sentence from a source, the child visualises the letter form, assigns meaning to it, manipulates the writing tool with motor control and eventually produces the written work (29).

VMI refers to the ability to coordinate visual information with a motor response and is the best predictor of handwriting legibility (14, 29, 42, 48–50, 56–58). VMI is tested widely using the Beery-Buktenica Developmental test of VMI (59). VMI in the Beery-Buktenica Developmental test is assessed by drawing simple or complex geometric forms.

On the other hand, the influence of visual perception (the ability to make sense of what is seen) on handwriting is unclear (56, 60, 61). Research on hand-eye coordination also has not indicated a strong relationship with handwriting (24, 29). However, Kaiser et al.’s (62) findings demonstrated that hand-eye coordination associated with VMI predicts handwriting quality.

### Fine Motor Skills

Fine motor skills in handwriting refer to finger movements coordinated with muscle movements in the wrist, elbow and shoulder to control a writing tool to produce text or writing (12, 29, 60, 63). Fine motor skills are related closely to handwriting (28, 29, 48, 54–56). According to Dinehart (64), early fine motor skills may even be useful in determining readiness for school. Many occupational therapists incorporate fine motor skills in their assessment of and interventions with clients who have handwriting difficulties (28, 29, 42, 43, 64, 65). Four fine motor skills that are correlated significantly with handwriting are identified, namely, as i) in-hand manipulation (finger functions); ii) fine motor precision; iii) manual dexterity and iv) motor planning.

In-hand manipulation (3, 14, 29, 43, 50, 55, 56, 65, 66) refers to the process of adjusting objects within the hand (54). During handwriting, the writer moves a writing tool with fingers and adjusts it to write. Some researchers have described in-hand manipulation as finger functions in handwriting studies (14, 67). In-hand manipulation may be examined through the fingers’ rotation, shift and translation (29, 54). Berninger and Rutberg (67) found that a finger succession task was the best predictor of handwriting and writing skills among other five-finger tasks used in their study. They suggested that the finger succession task was also the best measure of motor planning. Motor planning in handwriting is the ability to plan, sequence and execute letters in words (42), and it is correlated positively with handwriting legibility (29).

During handwriting tasks, the student stabilises the writing paper with their nonwriting hand, demonstrating bilateral integration ability, which is correlated with handwriting (29, 43, 45). Fine motor skills, such as motor precision and high-coordination (dexterity) when using a writing tool, also were found to be correlated positively with handwriting tasks (29, 50, 55, 68).

### Gross Motor Skills

Gross motor skills mainly refer to postural control during handwriting tasks (69). Body posture influences the efficiency of handwriting production (45) because the trunk’s stability allows the writer to adjust their posture to accomplish tasks that require fine motor skills, such as handwriting (60). Cheng et al.’s (70) study further confirmed that lower body stabilisation was important in providing support to the body during writing for children with cerebral palsy.

According to Erhardt and Meade (60), good posture entails:

“…sitting with hips at 90° angle and feet stabilised on the floor, good pelvic and spinal alignment, cervical control for downward visual gaze and shoulder integrity for arm and hand control” (p. 199).

Studies by Blote et al. (71) and Sassoon et al. (72) found a weak correlation between writing posture and handwriting quality, but a strong correlation between writing posture and handwriting speed (63, 69).

Kinaesthesia is the awareness of movements in our body, and proprioception is the sense through which we perceive the position of joints in our body (73). Cornhill and Case-Smith (29) relate kinaesthesia to the level of pressure applied to the writing tool, and the ability to write within boundaries. Kinaesthesia has demonstrated a significant correlation with handwriting legibility (29, 74). Schneck (74) found that kinaesthesia influences handwriting by influencing pencil grip, but Tseng and Murray (49) reported conflicting results. The proprioception sense did not correlate to writing legibility (75), but Schneck (74) found a possible relationship between children’s pencil grip and proprioception.

## Ergonomic Factors

Ergonomic factors also play an important role in handwriting performance (36, 45, 63, 76), but they have received less attention (36). Ergonomic factors relate to the design of certain tools, machines, systems, tasks, jobs and environments to optimise them for human use (45). The ergonomic factors in handwriting include pencil grip, the positioning of pencil and paper, consistency of pencil grip and pencil position, and pressure applied to the writing tool. Many occupational therapists focus on pencil grip in their intervention (42), as they believe that immature pencil grip may result in difficulty controlling finger movements while writing (43). Extant research has found that most poor writers possess an immature pencil grip (74, 77), but other studies also have demonstrated that pencil grip is not related to handwriting legibility and speed (36, 50, 63, 76). Only one study, by Schneck (74), found a positive relation between handwriting and pencil grip.

Pencil positioning and pencil grip consistency also are correlated highly to handwriting (36, 45). In addition, Parush et al. (45) reported other correlated ergonomic factors, including pressure consistency and paper positioning. However, it should be noted that although these ergonomic factors are associated with handwriting, no causal relationships have been established (45).

A notable study by Dennis and Swinth (78) examined the relationship between handwriting endurance and pencil grip and found that pencil grip did not affect task endurance, but that task length affected handwriting legibility.

## Orthographic Factors

Orthography is the graphic representation of spoken language, and graphic forms in alphabetic orthography contain phonological units (79). Three orthographic effects that are related to handwriting have been identified — letter knowledge, orthographic coding and syllable-size processing units — so it would be relevant to include these orthographic effects in the Malay language handwriting conceptual framework.

### Letter Knowledge

According to Fears and Lockman (80), letter recognition influences early handwriting. Letter knowledge refers to children’s familiarity with letter shapes, names and corresponding phonemes (81). Piasta and Wagner (82) presented five letter knowledge outcomes, namely: i) letter-name knowledge; ii) letter-sound knowledge; iii) letter-name fluency; iv) letter-sound fluency and v) letter writing. Letter-name knowledge and letter writing are highly correlated (83). Children need to learn the letters in the alphabet before they can start to learn writing.

### Orthographic Coding

Berninger et al. (84) defined orthographic coding as retrieving letter forms from memory to write. Berninger et al. (84) administered a modified orthographic coding task to children in each grade to examine the ability to retrieve orthography from memory. The children needed to identify whether a previously shown card contained a whole word, single letter or letter cluster. Beginning writers require the ability to store and retrieve a single letter, a cluster of letters or a whole word from memory during writing; therefore, automatisation of orthographic (letter, cluster and word) coding/retrieval from memory will increase handwriting speed (58). The findings from Berninger et al.’s study (84) also revealed a pattern as to how children progress from relying on whole word coding to letter and letter cluster coding as they begin to grasp GPC knowledge. The error analysis from orthographic coding tasks suggested that orthography-phonology correspondence may be related to orthographic coding. On the other hand, Weintraub and Graham’s study (14) did not find a correlation between orthographic processes (letter writing, orthographic speed test, expressive orthographic coding) and handwriting legibility.

### Syllable-Size Processing Units

Kandel et al. (33) found consistent dysfluency at the grapheme and syllable boundary in French children’s handwriting. It was suggested that cognitive load during the handwriting process caused this dysfluency, and they concluded that children use both grapheme units and syllable units as inputs to the motor system during handwriting tasks. As the Malay language is predominantly multi-syllabic, the question arises as to whether this dysfluency also occurs in Malay handwriting. Another interesting study, by Kandel and Valdois (85), measured the amount of gaze lift during a task that required copying words and nonwords among children in first through fifth grades, suggesting that students engaged in sub-lexical processing with unfamiliar words. Younger children who lacked sufficient orthographic information to copy whole word units displayed more gaze lift, but older children were able to copy a bigger word chunk (e.g. syllable and whole word), indicating that syllable segmentation processes have a grade-level effect on handwriting.

Kandel and Valdois (85) suggested that the emergence of syllable-processing units in their study was due to the French language’s orthographic depth — an intermediate level of transparency that encouraged the adoption of syllable-size processing units. A study on Spanish orthography — a more transparent orthography than French — yielded the same results (86). Other research also found morpheme-size processing units that regulated handwriting tasks (87). The question arises as to what role syllable-size processing units play in Malay language handwriting, considering that the language is predominantly multi-syllabic.

In another study, by Kandel et al. (88), they compared the results from French children copying words that are mono-syllabic phonetically, but bi-syllabic orthographically, and words that are bi-syllabic both phonetically and orthographically. Their findings indicated that children use orthographic, rather than phonological syllables as processing units to plan words mentally before they write.

In addition, the syllable structure was found to constrain motor production in handwriting within both French and Spanish orthographies (89). Lambert et al. (34) examined the writing of two- to four-syllable words and nonwords among adults and found that during the nonword task, syllable-size chunks were observed. According to Graham et al. (11), the act of chunking the letter into syllable-size processing units reduces attention and memory demands, thereby allowing for higher-level writing processes (11).

## Cognitive Factor

According to Hayes and Berninger (23), writing requires several cognitive components that operate at different levels during the writing and composing processes. Three cognitive components involved in handwriting are identified from the literature, namely working memory, long-term memory and executive attention or working memory capacity (18, 19, 23, 43, 53, 90–96). These cognitive factors closely interact during performance of complex tasks, such as handwriting.

**Figure 1:**
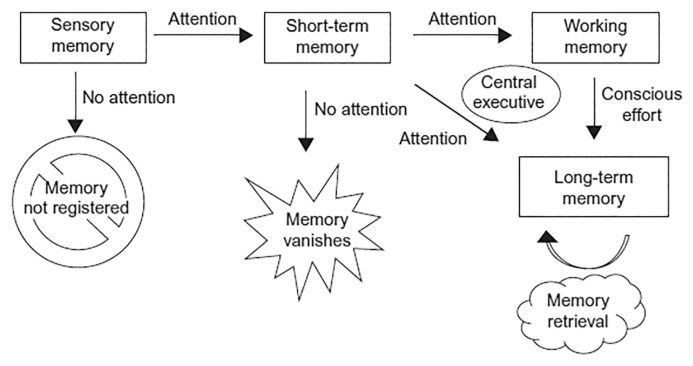
Progression of memory (98)

During transcription (translating language presentations into written words), substantial attention is required. In this process, working memory retrieves related information (e.g. letter forms, letter sequences, letter writing, etc.) from long-term memory and maintains the information until handwriting is executed (97). Figure 1 illustrates the connections between working memory, attention (working memory capacity) and long-term memory (98).

### Working Memory

Handwriting is a complex task that requires working memory (19), which is a temporary platform for storing, processing and retrieving information from long-term memory (53, 90, 93, 95). Typically, the terms ‘working memory’ and ‘short-term memory’ are used ambiguously in extant studies, and even interchangeably in many studies (99). Although there is overlap between both terms, as they share similarities, it depends on the task studied. However, ‘working memory’ is used instead of ‘short-term memory’ in this handwriting literature review because handwriting involves manipulation of information stored in the temporary platform for further processing.

### Long-Term Memory

According to Berninger et al. (17), handwriting not only involves the generation of letter representations in memory, but also retrieval of representations from memory. Therefore, poor memory impedes retrieval of letter forms from memory (100). Long-term memory functions as storage for information, and its duration and storage capacity is unlimited (98). The more knowledge writers possess in their long-term memory, the better their writing quality and fluency (23). In developing handwriting skills, a beginner will retrieve letter knowledge (in this case, alphabet letters) from long-term memory.

### Executive Attention (Working Memory Capacity)

Just and Carpenter (18) proposed a capacity model of working memory that was found to fit writing acquisition (96). Handwriting may strain working memory’s processing capacity, especially among beginning writers, because of its complex processes that involve the writer’s neuromotor development and the language’s orthographic characteristics (18, 89, 101).

Olive (101), in his review, presented an integrated model of the coordination of the writing process (cascading and parallel), which clearly communicated how working memory capacity affects coordination of the writing process. This also was demonstrated in Kandel et al.’s extensive research (33, 34, 85, 87–89) on the syllable processing units mentioned previously.

The central executive in Baddeley and Hitch’s model of working memory in 1974 (90) presents a function that resembles working memory capacity, as it governs the working memory system by allocating attention within working memory (e.g. distributing attention between multiple tasks), allowing for simultaneous input from different types of sensory information.

In addition, the executive function was found to be correlated highly with attentional control (91): ‘Attentional control has been conceptualised as executive functioning by neuropsychologists and as working memory capacity by experimental psychologists’ (94, p.1). Findings from McCabe et al. (94) suggest merging working-memory capacity with executive function as executive attention, considering that they both represent similar attentional control in performing complex tasks.

Therefore, working memory resources (attention) are shared among various processes involved in a task (19, 95, 96). An overload in working memory during handwriting tasks reduces retrieval performance, as attention is focussed on planning and performing motor components (19). This approach emphasises the role of automatisation of low-level processes, which free up capacity for high-level processes (95). Upon achieving automatisation of low-level skills in handwriting, writers can focus on high-level processes (1, 19, 53). Studies have demonstrated that the automatisation of handwriting progresses with grades and age (1, 3, 53, 102).

The Malay language’s bi- and multi-syllabic language characteristic might pose a challenge to writers, as a higher demand on executive attention is necessary (19). A three-syllable word, which is common in the Malay language, is longer than three-syllable English words. Therefore, rehearsal is needed to maintain these syllables in working memory and to avoid information decay of the syllables (30, 31). Although the high transparency and consistency of phoneme-grapheme correspondences in the Malay language may reduce some demand on attention resources and facilitate recall of motor production during handwriting preparation, constant rehearsal processes are needed to maintain multiple syllabic Malay words in working memory. We believe that the need to maintain (by constant rehearsing) these multiple syllables in a word in working memory would increase the demand for attentional resources; therefore, any effect from consistency in the language on cognitive load will be minimised. Thus, these divergent postulations lend credence to the need to study handwriting in relation to language characteristics.

## An Interdisciplinary Conceptual Framework of Malay Language Handwriting

**Figure 2:**
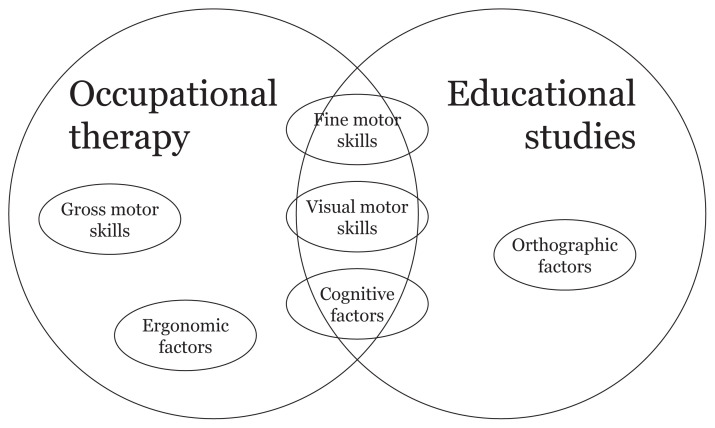
Mapping of handwriting factors from two disciplines

We now propose an interdisciplinary Malay language handwriting conceptual framework after analysis and synthesis of knowledge from the two major handwriting research disciplines. From the literature reviewed, four major factors that may influence Malay handwriting are identified, namely neuromotor development and ergonomic, orthographic and cognitive factors. Malay language characteristics may influence orthographic and cognitive factors directly, but they also may influence lower-level handwriting factors (neuromotor development and ergonomic factors), indirectly. For example, if a child is underdeveloped in neuromotor development, both execution of handwriting and the cognitive process during handwriting would be vying for more attention resources to perform handwriting. Many neuromotor development and ergonomic factors are derived from the occupational therapy discipline, whereas the education discipline provides most of the information on linguistic and memory factors. Neuromotor development factors include VMI, fine motor skills (in-hand manipulation, motor planning, bilateral integration, motor precision and hand coordination) and gross motor skills. Ergonomic factors primarily comprise types of pencil grip, pencil grip consistency and pencil positioning. Orthographic factors include letter knowledge, orthographic coding and syllable-size processing units. Finally, cognitive factors include working memory, long-term memory and executive attention. The primary knowledge sources of these four major handwriting factors are mapped and provided in Figure 2.

These four main factors are likely to contribute to individual differences in the Malay language handwriting process. An interdisciplinary framework that links knowledge between the two disciplines is presented in Table 1 and will provide a more coordinated and coherent reference to stimulate future handwriting research. From this framework, it is hypothesised that Malay language handwriting fundamentals comprise four major factors, along with sub-factors. Next, empirical research will be needed to reveal the actual factor structure of Malay language handwriting, each factor’s unique contributions to Malay language handwriting and the interlinkages among these factors and handwriting.

## Conclusion

This study proposed an interdisciplinary conceptual framework on Malay language handwriting to spotlight handwriting’s importance in academic competency. This framework will help develop empirical studies to answer pertinent questions about Malay language handwriting fundamentals, and these answers will form the basis for the design and development of assessment and intervention methods to address handwriting difficulties, to be applied by teachers as Tier 1 and Tier 2 interventions in inclusive classrooms. Finally, this paper spotlights orthography’s overlooked role in handwriting. The Malay language served as a case study for research into handwriting orthographies.

## Figures and Tables

**Table 1:** An interdisciplinary conceptual framework of Malay language handwriting

Task	Factors	Sub-factors	
Handwriting (legibility and speed)	Neuromotor development	VMI	
		Fine motor	In-hand manipulation
			Motor planning
			Motor precision and dexterity
			Bilateral integration
		Gross motor	Postural control

	Ergonomic	Pencil grip	
		Pencil and paper positioning/consistency	

	Orthography	Letter knowledge	
		Orthography coding	
		Syllable-size processing unit	

	Cognitive	Working memory	
		Long-term memory	
		Executive attention (working memory capacity)	
